# Novel genes exhibit distinct patterns of function acquisition and network integration

**DOI:** 10.1186/gb-2010-11-12-r127

**Published:** 2010-12-27

**Authors:** John A Capra, Katherine S Pollard, Mona Singh

**Affiliations:** 1 Gladstone Institutes, University of California, San Francisco, 1650 Owens St, San Francisco, CA 94158, USA; 2 Department of Computer Science and Lewis-Sigler Institute for Integrative Genomics, Princeton University, 35 Olden St, Princeton, NJ 08544, USA

## Abstract

**Background:**

Genes are created by a variety of evolutionary processes, some of which generate duplicate copies of an entire gene, while others rearrange pre-existing genetic elements or co-opt previously non-coding sequence to create genes with 'novel' sequences. These novel genes are thought to contribute to distinct phenotypes that distinguish organisms. The creation, evolution, and function of duplicated genes are well-studied; however, the genesis and early evolution of novel genes are not well-characterized. We developed a computational approach to investigate these issues by integrating genome-wide comparative phylogenetic analysis with functional and interaction data derived from small-scale and high-throughput experiments.

**Results:**

We examine the function and evolution of new genes in the yeast *Saccharomyces cerevisiae*. We observed significant differences in the functional attributes and interactions of genes created at different times and by different mechanisms. Novel genes are initially less integrated into cellular networks than duplicate genes, but they appear to gain functions and interactions more quickly than duplicates. Recently created duplicated genes show evidence of adapting existing functions to environmental changes, while young novel genes do not exhibit enrichment for any particular functions. Finally, we found a significant preference for genes to interact with other genes of similar age and origin.

**Conclusions:**

Our results suggest a strong relationship between how and when genes are created and the roles they play in the cell. Overall, genes tend to become more integrated into the functional networks of the cell with time, but the dynamics of this process differ significantly between duplicate and novel genes.

## Background

Large-scale genome sequencing efforts have made it increasingly possible to study the genetics of species divergence on a genome-wide scale. Comparing the complete genomes of many closely related species in the context of well-resolved phylogenetic trees provides clues about the genomic events and evolutionary processes that generate functionally-relevant differences between species. Several studies have identified lineage-specific differences in the gene sets of recently diverged species in many clades [1-4], and these observed differences often contribute to functional divergence between species [5-7]. Understanding the origin and function of new genes is critically important to deciphering the evolution of cellular networks and genomes; however, previous analyses have not taken into account the different evolutionary mechanisms that can produce new genes.

New genes are created by a variety of processes, including gene duplication, domain shuffling, incorporation of mobile elements, gene fission and fusion, and *de novo *acquisition (reviewed in [8]). Gene duplication has long been appreciated as an essential source of new genes and genetic novelty [9]. Whereas duplicate genes typically retain significant homology to their parent genes, evolutionary mechanisms like domain shuffling and gene fission and fusion can generate genes with new combinations of pre-existing functional elements [8,10]. Moreover, *de novo *gene creation from non-coding sequence is increasingly recognized as an important source of new genes. Examples of recent *de novo *gene creation have been found in fungi [11,12], flies [13-15], and mammals [6,16,17] - with estimates that as many as 12% of new genes in fly and 6% in human were created from non-coding sequence. Surprisingly, as more genomes have been sequenced, the prevalence of 'orphan' genes, with little to no similarity to other known genes, has not decreased; they still represent around 10-20% of all known genes [18-21].

The diversification of gene function after duplication and its role in the creation of lineage-specific phenotypic differences has been given substantial attention in genome-wide studies [3,7,21-27]. Duplication can occur at dramatically different scales, from the duplication of a relatively short segment of the genome to whole-genome duplication (WGD). Recently, several studies have demonstrated the relevance of the scale of a duplication that copies a gene to its functional consequences [28-30]. For example, the Baker's yeast, *Saccharomyces cerevisiae*, underwent an ancient genome duplication [31], and it has been proposed that the WGD was instrumental in enabling the highly fermentative lifestyle that characterizes *S. cerevisiae *and its close relatives [32,33]. Paralogs in *S. cerevisiae *generated by the WGD are also more likely to share interaction partners and have similar biological functions than duplicates created by small-scale events [34]. It has been argued that duplication of a single gene that takes part in a functional complex may create a stoichiometric imbalance [35]. This imbalance could increase the pressure for this duplicated gene to diverge in function and interactions, especially as compared to genes duplicated in a large-scale event such as a WGD that potentially maintains the balance within the complex [36].

Genes created by means other than duplication of a complete gene - which we refer to as *novel *genes - are likely to be under different evolutionary pressures than those created by either small-scale or large-scale duplication. Novel genes' sequences may not initially be functional or structurally well-formed. In contrast, duplicate genes are typically born with the ability to fold into stable structures with established functions and the potential to interact with their ancestor's interaction partners. The fate and function over time of novel and duplicate genes may reflect and reveal the effects of these differences. However, the functional evolution of novel genes soon after creation has not been broadly characterized.

To explore how these different evolutionary processes shape functional innovation in a lineage, we developed a computational approach that integrates various functional attributes of genes, including length, annotated functions, essentiality, and physical interactions, with a classification of genes into groups reflecting their mechanism of origin and time of creation. Because of the challenges associated with accurately inferring a gene's mechanism of origin and age [37,38], we considered several complementary computational approaches for categorizing the genes, and focused on broad statistical trends. Applying our analysis pipeline to *S. cerevisiae*, we performed a systematic, genome-wide comparison of the dynamics of function acquisition and interaction network integration between novel and duplicate genes. We found evidence of a strong relationship between the context of a gene's origin and its integration into the functional networks of the cell. Both novel and duplicate genes, on average, appear to gain interactions and functions over time, but the rate of this gain is more rapid for novel genes. A dramatic gain in gene length was observed with age for novel genes; this suggests that the integration of additional sequence elements over time may contribute to this increase in function. Overall, our findings argue that both the time and mechanism of creation are relevant to understanding how genes' functions evolve, and that differences in gene creation mechanisms are reflected in the fate and function of the genes they create.

## Results

### The classification of genes by age and origin

**Figure 1:**
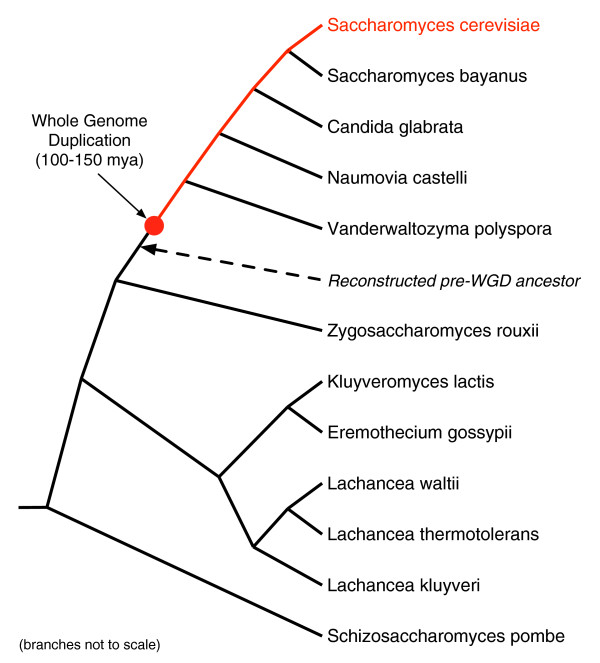
**Yeast species tree**. We analyzed functional attributes and interactions of genes gained since the whole-genome duplication (red circle) along the path leading to *S. cerevisiae*. We assigned genes in *S. cerevisiae *to one of three age groups, pre-WGD, WGD, or post-WGD. The assignment was based on the recent reconstruction of the gene content of an ancestral pre-WGD yeast, which was derived from an analysis of the sequence similarity and synteny of genes in the listed species [39]. An analysis using additional, more specific age groups is presented in Section S2.2 in Additional file 1.

We predicted the time of creation and mechanism of origin for each gene in *S. cerevisiae *(see Methods for details). Briefly, we classified all genes in *S. cerevisiae *into one of three 'age' categories: pre-WGD genes that were present before the WGD event approximately 100-150 million years ago; WGD genes that were duplicated by the WGD and maintained; and post-WGD genes that have appeared since the WGD. Genes present before the WGD (pre-WGD) will also be referred to as 'old', while those created since the WGD (post-WGD) will, in comparison, be referred to as 'young' or 'recently created' (even though they may be 100 million years old). The classification of each gene was based on its presence or absence in a curated reconstruction of a pre-WGD yeast ancestor from Gordon *et al*. [39]. In that work, sequence similarity and synteny were used to trace by hand the evolutionary history of each gene in 11 fully sequenced yeast species (Figure 1) and reconstruct the gene content and order of the *S. cerevisiae *ancestor immediately before the WGD.

Next, we assigned *S. cerevisiae *genes to origin categories, duplicate or novel. Since predicting the mechanism of origin for a gene is a challenging task, we used several approaches. The first is a family-based approach that considers the presence or absence of paralogous genes in the genome. Genes with at least one paralog in *S. cerevisiae *were assigned to the duplicate category. Genes with no paralogs were assigned to the novel category. The evolutionary families of homologous genes used in this classification were predicted using the Jaccard Clustering algorithm from the Princeton Protein Orthology Database (PPOD) [40,41]. As an alternative origin classification, we considered gene trees and orthogroups predicted by Synergy [42], a computational method that uses gene sequence similarity and synteny to reconstruct genome-wide evolutionary histories of gene families. While gene loss and rapid evolution can confound both methods of classification (see Discussion), in each case, the duplicate category contains genes likely to have been created by a duplication of a complete gene, and the novel group contains genes likely created by one of the non-duplicate mechanisms that yield genes of novel sequence and structure. For ease of exposition, we report results from the evolutionary family-based classification in the main text. In Additional file 1, we show that our main conclusions hold based on the Synergy-based origin classification scheme, and include several additional controls, including the exclusion of harder to classify genes in the dynamic subtelomeric regions. A fuller description of the classification process is included in the Methods.

Considering the age and family-based origin categories together, we predicted 1,434 pre-WGD/duplicate, 2,696 pre-WGD/novel, 1,087 WGD/duplicate, 314 post-WGD/duplicate and 239 post-WGD/novel genes. No novel genes were created by the WGD, so the empty WGD/novel group is ignored. Only non-dubious genes, as annotated by the *Saccharomyces *Genome Database (SGD) [43], were considered, so as to eliminate sequence regions that resemble genes, but that are not actually translated and transcribed (for example, pseudogenes and spurious predictions from gene finding programs). This classification of genes in provided in Additional file 2[44].

### Functional properties of young novel and duplicate genes

As a first step in the investigation of the influence of gene age and origin on function, we analyzed the age/origin gene groups with respect to four attributes that reflect different aspects of gene function. First, we considered the length of the protein encoded by a gene. Protein length imposes physical constraints on the number of functional domains it can contain. Second, we measured the fraction of each protein's amino acids that are predicted to take part in a Pfam domain. Protein domains are the fundamental units of protein structure and function, and protein domain families from Pfam [45] provide a view of the units that enable proteins to function. Third, we report the fraction of genes in each age/origin group that are known to be essential. Essentiality, as determined by the viability of a deletion mutant [46,47], gives an indication of the importance of the gene to the species. Fourth, we calculated the fraction of genes that have been annotated with terms from each of the Gene Ontology (GO) functional hierarchies [48]. GO annotations reflect what is currently known about a gene's function.

**Figure 2:**
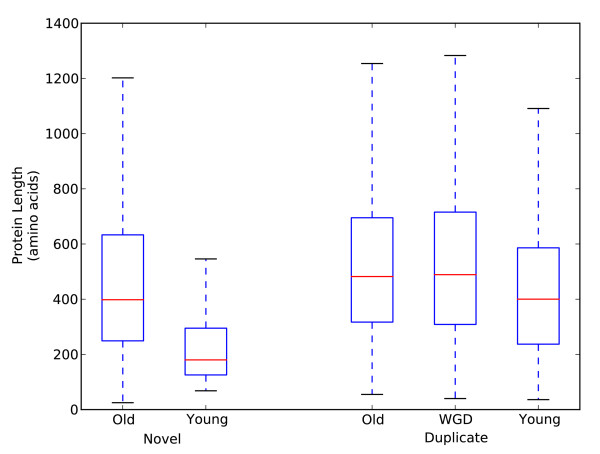
**Protein length distribution by age and origin**. The box plots summarize the distribution of sequence lengths (in amino acids) of the proteins with interactions found in each age/origin group. Young proteins are shorter than older proteins, and young novel proteins are significantly shorter than all other groups, including young proteins created by duplication.

Using these four functionally relevant gene properties, we compared genes across both time of creation and mechanism of origin (Table 1, Figure 2). The significance of differences in these functional attributes between the age/origin groups was assessed by a Mann-Whitney *U *test, and all differences discussed in this section are significant at the 0.05 level.

**Table 1:** The coverage of gene groups by different sources of functional information

Origin	Age	Number of genes	Pfam coverage^a^	Fraction essential^b^	GO MF coverage^c^	Fraction with interactions^d^
Novel	Old	2696	0.36	0.30	0.67	0.77
	Young	239	0.12	0.00	0.22	0.25
Duplicate	Old	1434	0.57	0.30	0.88	0.81
	WGD	1087	0.45	0.08	0.76	0.72
	Young	314	0.53	0.02	0.60	0.55

^a^Pfam coverage: average fraction of protein length covered by Pfam domains; ^b^fraction essential: fraction of genes in each group that are essential; ^c^GO MF coverage: fraction of genes with Gene Ontology (GO) Molecular Function (MF) annotations; ^d^fraction with interactions: fraction of genes with interactions in the protein physical interaction network. Each statistic is calculated over those genes whose proteins have known physical interactions.

#### Genes in each age/origin group differ in their properties

Comparing the properties of proteins corresponding to the genes across age and origin groups revealed several general trends (Table 1, Figure 2). Young proteins are shorter, have fewer known functions, and are less essential than their older counterparts. This difference between young and old proteins holds when considering both novel and duplicate genes separately. However, the differences between old and young duplicate genes in each of the functional properties, except for essentiality, are much less dramatic than among novel genes of different ages. Within proteins of similar age, there are also marked differences. For nearly all properties considered, the novel proteins have less evidence of function than duplicate proteins from the same age group; they have a lower fraction of coverage by Pfam domains, are less likely to have known annotations from GO, and have fewer proteins with known physical interactions. The one exception to this pattern is essentiality among the old genes; the old novel genes are as likely to be essential as the old duplicate genes. However, the young novel genes are less essential than the young duplicate genes. In general, the differences between the older genes of different origin are less dramatic than those between the young novel and duplicate genes.

#### Young novel genes are particularly short, non-essential, and minimally annotated

Young novel genes are by far the most distinct group with respect to the properties analyzed in the previous section (Table 1, Figure 2). They are significantly shorter than young duplicate proteins; the proteins in these groups have median length of 180 amino acids (aa) and 400 aa, respectively. Young novel proteins are also less covered by Pfam domains (12% vs. 53%); this suggests that many young novel proteins are not simply rearrangements of pre-existing functional domains, but rather that they often consist of novel functional units. In addition, young novel genes are essential less frequently than young duplicates (0% vs. 2%), and are much less likely to have GO Molecular Function annotations (22% vs. 60%). As suggested by these results, young novel genes are also significantly different from older novel genes. The median length of their corresponding proteins is less than half as long (180 aa vs. 398 aa); their amino acids are on average three times less likely to participate in known Pfam domains; they are not essential (0% vs. 30%); and a smaller fraction have annotations as compared to old novel genes (22% vs. 67%). Thus, four largely independent lines of evidence suggest that novel and duplicate genes have distinct functional properties and that young novel genes have fewer functional abilities than old.

We note that differences in the number of annotations, according to GO, between the gene groups could be the result of a bias in the amount of study genes of different groups have received, rather than differences in the number and character of the functions actually performed by the genes in the group. The three additional functional attributes we considered are less subject this potential bias. Sequence-derived properties such as length and coverage by Pfam domains (we include Pfam sequence motifs corresponding to 'domains of unknown function'), as well as the fraction of tested genes found to be essential, do not depend on the number of experiments carried out on a gene.

### The physical interactions of novel and duplicate proteins

Proteins function by interacting with one another and with other molecules in the cell. Thus, a protein's physical interactions and its integration into the topology of the interaction network provide additional evidence about its functions, how they are accomplished, and their overall importance to cellular functioning [49-56]. We explored whether the proteins corresponding to genes in each of the age/origin groups differ with respect to their frequency of physical interactions, their relative location within the yeast protein interaction network, and the identity of their interaction partners.

We constructed a protein-protein physical interaction (PPI) network for *S. cerevisiae *using interaction data from small-scale experiments and high-throughput studies as defined by the Database of Interacting Proteins (DIP) [57]. For each protein with known interactions, we quantified its integration into the yeast protein-protein interaction network by two graph-theoretic measures of the importance of a node within a network. The degree (or degree centrality) of a protein in the physical interaction network is the number of known interaction partners, and is a local measure of the topological centrality of the protein in the network. The betweenness centrality of a protein is the fraction of all shortest paths in the network that contain it, and is a global measure of the importance of a node within a network.

#### Young proteins are less integrated into the physical interaction network than older proteins

**Figure 3:**
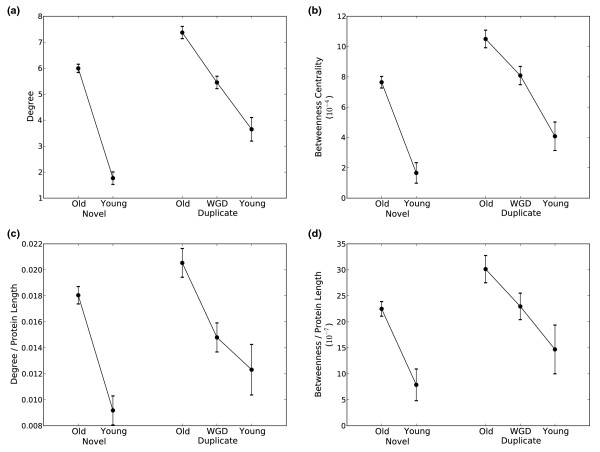
**The integration of proteins into the yeast protein-protein interaction network by age and origin**. Each pane gives the average (with standard errors) of a statistic that reflects the integration of proteins in each age/origin group into the yeast physical interaction network: **(a) **degree, **(b) **betweenness centrality, **(c) **degree divided by protein length, and **(d) **betweenness divided by protein length. Similar trends are seen in each plot. Young proteins are less integrated than older proteins, and duplicate proteins are more connected than novel proteins. Overall, the young novel proteins are significantly less integrated than all other groups.

Both young novel and young duplicate proteins have fewer interactions than their older counterparts (Figure 3a). There is a clear increase in degree with age, and in general, proteins created by the WGD are found to have degree between the older and younger groups. A similar increase with age is observed among the groups when considering betweenness centrality (Figure 3b). Only proteins with known interactions are considered in this analysis, but we also note that a considerably smaller fraction of young proteins have known interactions than the old proteins (Table 1). The differences in the network integration of young and old proteins would be even more extreme if all proteins were included.

We considered three additional related tests that also support the finding that young proteins are less integrated within cellular networks. First, since a protein's size places physical limits on the number of interactions it can simultaneously maintain with other proteins and molecules [58], we also normalized the degree and betweenness of each protein by its length (Figure 3c, d). The increase in interactions with protein age is still present after this normalization, though it is somewhat reduced. Second, since essential proteins have been found to participate in more interactions than non-essential proteins [59,60] and older proteins are more frequently essential (Table 1), we repeated the analysis excluding essential proteins and find the same relationship between the age of a protein and its network context (Section S1.1.3 in Additional file 1). Third, since the presence of interactions in the network derived from small-scale studies could introduce a bias toward interactions involving well-studied proteins, we repeated the analysis including only interactions determined by high-throughput studies, and found that young proteins are also less integrated than older proteins in the network resulting from only high-throughput experiments (Section S1.2 in Additional file 1).

#### Novel proteins are less central in the network than duplicate proteins of the same age

Comparing the distributions of degree and betweenness centrality between groups of proteins based on origin reveals that duplicate proteins are more centrally located in the network than novel proteins of the same age (Figure 3). The novel proteins have on average both lower degree and lower betweenness centrality than their duplicate counterparts. Because young novel proteins are significantly shorter than all other types (Figure 2), the large difference in raw degree between the young novel proteins and young duplicate proteins is reduced when normalized by protein length (Figure 3c, d). Nonetheless, the young novel group is still more peripheral than any other group, even after length normalization.

#### Proteins preferentially interact with proteins of same age and origin

**Figure 4:**
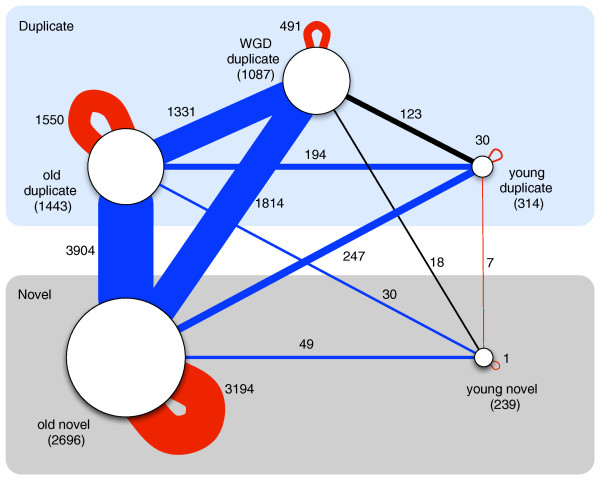
**Schematic view of the interactions between different age/origin groups in the yeast protein physical interaction network**. The age/origin groups are represented by nodes, with node size illustrating the group size, and the number of genes in each group in parentheses. The number of interactions between proteins of each group are given on the edges between group nodes, and are reflected in the edge width. The color of an edge indicates whether significantly more (red), fewer (blue), or an expected number (black) of interactions are observed in the network (see Figure 5).

The analysis of the previous sections considered the number of interactions for proteins in the physical interaction network. The identity of a protein's interaction partners also gives information about how it functions. Each interaction in the network can be classified based on the group membership of the interacting proteins. Figure 4 shows a schematic view of the yeast protein-protein interaction network that gives the number of interactions between different age/origin groups.

**Figure 5:**
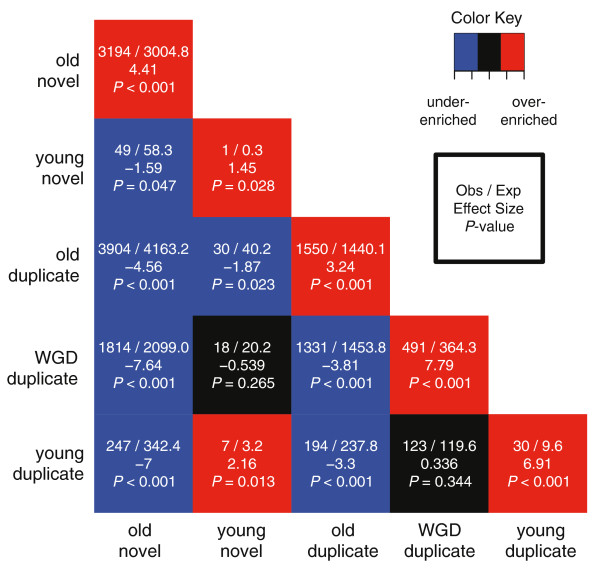
**Significance of interaction preferences by protein age and origin**. Proteins preferentially interact with other proteins of the same age and origin. The top line in each box gives the number of observed interactions of each type and the number expected based on 1,000 random networks. The middle line gives the effect size (Glass's Δ), and the bottom line gives an empirical p-value for the difference between the observed and expected (Methods). Squares in red indicate significantly more interactions than expected; blue indicates significantly fewer than expected; and black indicates no significant difference. The red trend across the diagonal reflects the significant preference for proteins to interact within their age/origin group. Nearly all of the off-diagonal interactions are significantly depleted. The only significant enrichment for interactions between proteins of different age or origin is among young proteins.

To investigate whether proteins demonstrate preferences in their interaction partners, we compared the number of each type of interaction observed in the actual network to the number expected to occur by chance in a random network that preserves the degree distribution of each protein group (Methods). The heat map in Figure 5 summarizes these results across pairs of age/origin groups with interactions between proteins in the same group listed along the diagonal. Proteins from all age/origin groups are significantly more likely to interact with proteins from their own group than expected by chance (*P *< 0.05 for each group). That is, proteins created by similar mechanisms at similar times preferentially interact with one another.

Interactions between proteins in different groups (that is, between proteins gained at different times or created by different mechanisms or both) generally occur significantly less often than expected (Figure 5 off-diagonal entries). For example, old novel proteins are significantly depleted for interactions with any group of duplicate proteins. The single instance of significant over-enrichment for interaction between different groups is among young proteins: young novel proteins interact more often than expected by chance with young duplicate proteins. We note that the significant preference to interact with proteins of the same group does not imply that proteins interact only with their group members. For example, the majority of interactions for both groups of young proteins are with proteins from other age/origin groups (Figure 4); however, the number of interactions observed within the groups is significantly greater than expected.

### The functions of young genes

We have shown that genes of different ages and origins differ with respect to their functional attributes and their context in the interaction networks of the cell. Since the creation of new genes may play a role in speciation and the development of novel phenotypes, the functions of young genes are particularly interesting. In this section, we investigate whether the differences in how novel and duplicate young genes are integrated into functional networks are reflected in their specific functions, and whether these gene gains can be tied to phenotypic differences in the corresponding species.

#### Young duplicate genes facilitate the processing and transport of sugars, while young novel genes do not exhibit enrichment for particular functions

Functional annotations from the GO hierarchies represent the current state of our knowledge of the functions of genes. Comparing the GO annotations observed in a subset of genes to the genome wide distribution of annotations can identify enriched functions that occur in the subset more than expected by chance [61].

The young duplicate set is enriched for genes that interact with the environment and that are involved in the processing and transport of sugars (*P *< 0.01 for all enrichments mentioned, see Tables S12-S14 in Additional file 1). For example, the enriched terms include carbohydrate transport, response to toxin, glucose transmembrane transporter activity, aryl-alcohol dehydrogenase activity and cell wall. This enrichment is notable given the distinctive fermentative abilities of *S. cerevisiae *and its close relatives (see Discussion). The innovation in functions related to carbohydrate processing appears to be focused in subtelomeric regions. When subtelomeric genes were removed from the enrichment analysis of young duplicate genes, these terms were no longer significantly enriched. However, the enrichment for many of the terms related to environmental response was maintained. The full list of enriched terms when excluding subtelomeric genes is given in Table S6 in Additional file 1.

In contrast, no significant enrichment for these or any other functions was found among the young novel genes with annotations. However, very few of these genes have GO functional annotations (Table 1). The terms observed among the young novel genes with annotations exhibit a wide variety of functions from transcription factor activity to flocculation.

#### Young novel proteins in their network context

**Figure 6:**
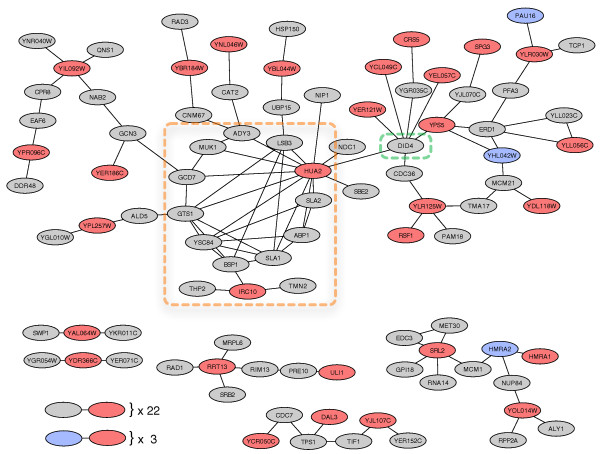
**Subnetwork of the yeast protein physical interaction network consisting of young novel proteins and their first-degree neighbors**. Young novel proteins are shown in red; young duplicate proteins are colored blue; and all others are gray. Nearly half of all young novel proteins are found in a single connected component (top of figure). The network module highlighted by the orange box consists of two young novel proteins interacting with a number of proteins involved in actin formation and processing. The protein highlighted in green is notable because it interacts or is within one neighbor of seven young novel proteins. Its function and those of nearby proteins suggest a role in membrane trafficking for several of the young novel proteins. Note that interacting protein pairs with no other interactions in this subnetwork are not listed. This visualization is based on the circular layout in CytoScape [99].

In order to better characterize the functions of young novel genes in the absence of direct experimental data, we took a detailed look at the context of the corresponding proteins in the yeast protein physical interaction network. Figure 6 shows the subgraph of the PPI network induced by the young novel proteins and their neighbors. Of the 239 young novel proteins only 59 have known physical interactions. These 59 proteins interact with a total of 89 proteins from other groups. GO terms observed among the protein physical interaction network neighbors of each young novel gene revealed a range of general functions, but again we did not observe significant patterns of enrichment as seen for young duplicate genes.

Remarkably, more than 40% of all young novel proteins with interactions (25 out of 59) are found in a single, relatively small connected component of 81 proteins. Within this connected component, there is a nearly complete subgraph which includes the young novel protein Hua2p (Figure 6). The proteins that make up this highly connected module are enriched for functions involved in aspects of actin assembly and regulation. These interactions led to the previous annotation of Hua2p with a possible role in actin patch assembly [62]. Hua2p has the most interactions of any young novel protein. In contrast, the young novel protein Irc10p has only three known interactions, and no known function. It is directly connected to the module that contains Hua2p, and two of its interaction partners have functions that suggest a potential connection to actin: Bsp1p links proteins to the cortical actin cytoskeleton [63] and Tmn2p is a transmembrane protein involved in filamentous growth [64]. Both Hua2p and Irc10p are present in only *S. cerevisiae *and its closest relative considered, *S. bayanus*. This suggests recent changes to processes involving actin due to the creation and integration of these young proteins.

Another notable feature in this 'young novel' subgraph is the old duplicate protein Did4p. It interacts with a large number of young novel proteins; seven of the young novel proteins are either adjacent to or one protein removed from it. Did4p is involved in the sorting of integral membrane proteins into lumenal vesicles of multivesicular bodies, and the delivery of newly synthesized vacuolar enzymes to the vacuole. It also plays a role in endocytosis [65-67]. Several of the proteins near Did4p in the network also have functions related to membrane trafficking and the endoplasmic reticulum. For example, Erd1p is a predicted membrane protein required for the retention of lumenal endoplasmic reticulum proteins [68], and the young duplicate protein YHL042Wp is a member of the DUP380 family thought to be involved in membrane trafficking [69]. This suggests that the nearby young novel proteins, such as Yps5p, which has similarity to cell membrane GPI-anchored aspartic proteases [70], and YCL049Cp, which localizes to the membrane [71], are likely involved in similar processes.

## Discussion

We introduced a computational approach for investigating the origin, function, and evolution of new genes by considering a phylogenetic classification of each gene in an organism within the context of its cellular interaction network. Applying this approach to *S. cerevisiae*, we have shown that there are significant differences between the lengths, interactions, and functional properties of groups of genes classified according to their ages (pre-WGD, WGD, or post-WGD) and their origins (duplicate or novel). Most notably, young novel genes are shorter, less annotated by a range of sources of functional information, and less integrated into experimentally determined physical interaction networks than other genes.

### Interpretations

Assuming that the early evolutionary history of older genes is reflected in modern young genes, our findings suggest that on average both novel and duplicate new genes tend to gain interactions over time. However, the dynamics of this gain appear to differ between genes based on their mechanisms of origin and context. In particular, novel genes start with far fewer annotated functions and interactions than duplicates. This is not surprising as duplicate genes often arise with the ability to interact with their ancestors' interaction partners, and their structures already have established functions in the cell. On the other hand, non-duplicate genes are likely created by evolutionary processes that generate novel sequences that initially may not be fully functional. The differences in length, interactions, and functions we identified between old and young novel genes are more pronounced than those uncovered between old and young duplicated genes. This suggests that novel genes may experience a more rapid gain in function over time.

The increase in novel gene length with age provides one possible explanation for the gain in interactions and functional capabilities: the incorporation of additional sequence elements. The integration of mobile genetic elements and surrounding sequence via mutation of start and stop codons could be responsible for this lengthening. These phenomena have been documented in detailed analyses of the evolutionary histories of several recently created genes [8] and regulatory elements [72]. However, the increase in interactions with age is maintained when gene length is taken into account (Figure 3). This argues that the addition of new sequence is not entirely responsible for the gain of interactions.

The differences we have uncovered between genes grouped together by their predicted age and origin strongly suggest that both the mechanism and time of creation influence how newly created genes gain functions. Several previous studies support aspects of this hypothesis. For example, proteins with similar phylogenetic profiles - patterns of occurrence of homologous proteins in organisms across the evolutionary spectrum - have been shown to have similar functions [73], and the increasing relationship between age and degree has been observed previously [74]. A very recent sequence-based analysis found that young genes in eukaryotes experience more variable patterns of selection than older genes with homologs in bacteria [75]. Our integration of predictions of age, origin, function, and interactions demonstrates that there are further distinct patterns in the functional evolution of newly created genes of different origin. The significant preference of proteins (both young and old) to interact physically with proteins in the same age/origin group (Figure 5) suggests that genes with similar evolutionary origins and histories are more likely to gain and participate in similar functions, perhaps reflecting adaptations in those functions during particular phases of the organism's evolution. The enrichment for a small number of specific functions in young duplicates and the presence of many young novel proteins in a single, small, connected subgraph of the PPI network are consistent with this hypothesis of coordinated integration.

The relevance of the context and time of creation to gene function and interactions also suggests a potential driving force for the modularity observed in protein interaction networks (see, for example, [76,77]). While several theoretical network evolution models that incorporate gene duplication and the subsequent gain and loss of interactions yield networks with similar properties to observed networks [55,78,79], recent work has identified subtle attributes of protein-protein interaction networks that cannot be explained by gene duplication and divergence alone [54,56]. The distinct patterns we identified in the interactions of genes created by mechanisms other than duplication suggest that modeling other types of gene creation will also be important in understanding the evolution of cellular networks.

### New genes and novel phenotypes

The creation of genes may allow a species to adapt to new environments. One of the major distinguishing characteristics of *S. cerevisiae *and its close relatives from other yeasts is the tendency to ferment glucose and accumulate ethanol even in the presence of oxygen. It has been proposed that the development of this preference was enabled by the WGD [32,80]. The enrichment for functions related to carbohydrate transport among the young duplicate group suggests that as many as 40 of the genes gained after the WGD may also be involved in generating and refining this novel trait in *Saccharomyces *yeasts. For example, ADH2 (YMR303C), an alcohol-dehydrogenase known to be central to this ability [81], was created by a duplication after the WGD. In stark contrast, the young novel genes are not found to be associated with these processes or with adapting to any other changes in the environment, further highlighting the importance of origin to the acquisition of function.

A large number of young genes in *S. cerevisiae *lack any information about their function. In addition, there is evidence that many open reading frames (ORFs) currently classified as dubious may actually encode functional proteins [82]. The recent discovery that *MDF1 *is a *de novo *protein-coding gene likely involved in mating type adaptation provides a striking example of this potential [12]. This gene was not included in our analysis, because it was classified as a dubious ORF at the start of our study. It will be exciting to continue exploring the existence and function of newly created genes and their involvement in lineage specific traits. Our analysis of the protein interaction network context of young novel genes in *S. cerevisiae *provides a step in this direction by suggesting roles in actin processing and membrane trafficking for several uncharacterized genes.

### Controls and robustness

We have described a procedure for categorizing genes with respect to age and origin, and relating them to cellular function. Our overall conclusions in *S. cerevisiae *are robust to a number of modifications in our analysis pipeline.

Inferring the evolutionary history and origin of a gene is an area of active research. Genes with fully traced evolutionary histories reveal complex series of events that can dramatically alter a gene's sequence and context in the genome over time [8]. Current methods for ancestral reconstruction cannot always accurately perform the basic inferences involved in these analyses, such as cross-species ortholog prediction [37] and the determination of the original copy after a duplication [38], much less trace more complex evolutionary events that may fuse, rearrange, and remove parts of genes. To account for these challenges, we considered different methods for predicting the origin of a gene. Though they did not always agree on specific predictions (Figure S1 in Additional file 1), they produced categorizations that are enriched with genes of the appropriate origin and amenable to statistical analysis. Indeed, our main conclusions hold across several different prediction methods (Section S1.1 in Additional file 1).

Bias toward the study of older, evolutionarily conserved genes could magnify the patterns of difference we observed between young genes and old genes. We accounted for this possible bias by confirming our conclusions on data from sources that are not based on small-scale experimental analyses. For example, as noted earlier, the significant increase in interactions with age also holds when considering interaction networks built from only high-throughput studies. Similarly, the small number of GO annotations for young and novel genes is supported by similar patterns in length, coverage by Pfam domains, and essentiality, each of which is less subject to bias. Pseudogenes and other spurious predicted genes also have the potential to confound our analysis. To limit their effect on our conclusions, we left all dubious ORFs (as defined by SGD) out of the analysis, and confirmed our results on the set of genes for which the corresponding proteins are known to participate in protein-protein interactions. This provides strong evidence that the genes considered are transcribed and translated.

### Future work

In the future, it should be possible to apply our procedure, with appropriate modifications, to other lineages. The large number of genomes available for primates and placental mammals together with a large-scale human physical interaction network [83] make human a promising future target.

Another avenue for further research is to consider more detailed categorizations of gene age and mechanism of origin. In the near future, a more fine-grained temporal analysis will likely be possible as more genomes are sequenced and computational methods for reconstructing evolutionary histories improve. As a first step, we considered a more specific division of gene age in which we distinguished the old (pre-WGD) genes into those created prior to the divergence of *S. cerevisiae *and *Schizosaccharomyces pombe *and those created after this divergence, but before the WGD [84]. Results were similar on this partition of the genes, and genes in the temporally intermediate group exhibited patterns that fell between those of younger and older genes, supporting our conclusion that genes, on average, gain functions and interactions over time (Section S2.2 in Additional file 1).

The different rates of network integration and functional gain we observed suggest that a more detailed analysis of the patterns and functional impact of specific mechanisms of gene gain and evolution could be fruitful. The novel gene groups contain genes created by a number of non-duplicate evolutionary mechanisms. Grouping these non-duplicate genes was necessary for our statistical analysis, because the absolute number of young genes is relatively small. However, the evolutionary forces acting on genes of *de novo *origin are likely to be very different from those originating from the rearrangement of existing domains, as the latter are more likely to be immediately associated with certain cellular functions and to have specific protein interactions. Depending on the lineages studied, we could divide this group of genes further. In prokaryotes, for example, it would be possible to investigate the integration of genes gained by lateral gene transfer into interaction networks.

## Conclusions

We expect that further characterization of recently created genes in organisms across the evolutionary spectrum, as well as a deeper understanding of the evolutionary mechanisms that generate and shape them, will play a central role in our understanding of the genetic basis of lineage-specific traits and adaptation.

## Materials and methods

### Data

Raw sequence data and annotations for the *S. cerevisiae *strain S228C genome were downloaded from the SGD on October 18, 2009 [85]. The reconstructed evolutionary history between *S. cerevisiae *and a pre-WGD ancestor derived by Gordon *et al*. [39] was downloaded from the Yeast Gene Order Browser (YGOB) Version 3 [86,87]. Predicted gene families and the corresponding homologous proteins were downloaded from the Princeton Protein Orthology Database [40,41] on October 18, 2009. The PPOD database includes predictions from OrthoMCL [88], MultiParanoid [89], and a Jaccard clustering-based approach. For the classification of subtelomeric genes not included in YGOB reconstruction, sequence alignments of *S. cerevisiae *proteins with predicted orthologs from seven related fungi were downloaded from SGD on November 24, 2009 [85].

Physical interaction data were extracted from the January 26, 2009 release of interactions in the Database of Interacting Proteins (DIP) [57]. In exploring the robustness of our conclusions, we also considered the physical interaction networks of Kim and Marcotte [54], which were extracted from BioGRID [83]. For the DIP network, proteins with more than 50 physical interactions were iteratively filtered so as to remove experimental artifacts due to 'sticky' proteins. The networks used by Kim and Marcotte [54] were filtered as described in Batada *et al*. [90]; this produced networks that were easily divided into a literature-curated interaction set and a set determined by high-throughput experimental methods. The reported conclusions hold on all of these networks (Section S1.2 in Additional file 1), suggesting that bias in the study of certain types of interaction is not responsible for the patterns observed.

We considered several sources of functional information about genes and proteins. First, the essentiality of a gene was taken from the viability data reported in SGD [47,85]. This includes data from a high-throughput screen of knockout mutants of nearly all ORFs in *S. cerevisiae *[46] and many small-scale studies. We considered a gene essential if it was found to be essential in any of the studies. Knowledge of the function of a protein was taken from the Gene Ontology database [48] maintained at SGD. The enrichment for functions among sets of proteins was tested using the GO:TermFinder tool [61]. The known domains present in each protein were taken from release 24 of Pfam-A [45]. The significance of observed differences in these properties between groups of proteins was assessed by a Mann-Whitney *U *test. Performing a two-way ANOVA on the groups also yielded similar results.

### Classification of genes by age and mechanism of origin

We assigned an age (pre-WGD, WGD, or post-WGD) and mechanism of origin (duplicate or novel) to each non-dubious *S. cerevisiae *ORF in SGD. We first describe the family-based scheme used in the main body of the paper, and then briefly describe alternate approaches that produced similar conclusions.

For each gene, a mechanism of origin was assigned based on the presence or absence of a paralog in *S. cerevisiae*. Genes found in a homologous family with more than one member in *S. cerevisiae *as defined by the Jaccard clustering method in PPOD [40] were classified as duplicate, and those without other family members in *S. cerevisiae *were classified as novel. Classification of genes into age groups was greatly facilitated by the recent reconstruction of the evolutionary history of *S. cerevisiae *to just prior to the WGD [39]. This reconstruction was carried out by hand and considered the sequence similarity and synteny of all genes in the species listed in Figure 1. If a gene was present in the predicted pre-WGD ancestor, it was assigned to the pre-WGD group. Duplicate gene pairs created by the WGD were assigned to WGD/duplicate. Though determining the scale of gene duplication is challenging [91], gene duplicates maintained from the WGD have a distinct signature when their genomic contexts in the reconstruction are compared. Only one copy will be present in the pre-WGD ancestor, and this gene will map to two distinct regions in *S. cerevisiae *that both maintain synteny to the ancestor [31,39]. Homologous families containing known WGD paralogs were merged. A gene whose ancestor is not found in the pre-WGD ancestor was assigned to post-WGD.

The YGOB's ancestral reconstruction does not include subtelomeric regions of the yeast genome because synteny breaks down in these highly species-specific regions. The subtelomeres are of considerable interest in this study because they contain nearly 300 genes - many with limited sequence similarity beyond *S. cerevisiae*. To extend our analysis to these genes, we considered the phylogenetic distribution of subtelomeric genes in the SGD's alignments of orthologs from seven fully sequenced species (*S. cerevisiae, Saccharomyces paradoxus, Saccharomyces mikatae, Saccharomyces bayanus, Saccharomyces kudriavzevii, Naumovia castellii, Lachancea kluyveri*). These alignments are based on the data and analysis of Cliften *et al*. [92] and Kellis *et al*. [93]; note that they include a different set of species than those used in the ancestral reconstruction. Genes with an ortholog in a species that diverged prior to the WGD were assigned to pre-WGD. All others were assigned to post-WGD. Since these predictions were obtained using a different strategy from those in the YGOB, we repeated our analysis excluding all subtelomeric genes. Our overall conclusions are maintained (Section S1.1.3 in Additional file 1).

Gordon *et al*. [39] analyzed 124 genes created since the WGD. The additional post-WGD genes in our classification come from two sources. As described above, we assigned nearly 200 subtelomeric genes that were left out of their reconstruction to the post-WGD group. The remaining additional genes were included in the data downloaded from the Yeast Gene Order Browser, but not considered in Gordon *et al*. Many of these genes were not classified as dubious by SGD and had physical interactions, so we included them in our analysis. Leaving out each of these groups of genes in turn and rerunning our analyses yielded similar results (Section S1.1.3 in Additional file 1).

The classification of sets of genes into age and origin groups is a challenging problem. We tested the sensitivity of our conclusions to several different family and evolutionary history inference methods. In particular, taking families from MultiParanoid [89] or OrthoMCL [88] (see Section 1.1.2 in Additional file 1). We also considered an origin classification based on gene trees and orthologous groups defined by the Synergy algorithm [25,42,94] for each gene in *S. cerevisiae*. If a gene had a predicted duplication at any point on the path to its ancestor in its gene tree or a homologous orthogroup, it was assigned to duplicate; otherwise, it was assigned to novel. This approach and the family-based method agreed on 76% of their predictions, and our main conclusions were maintained with this definition of origin. This supports our interpretation that, though gene loss and rapid evolution may introduce errors in individual classifications, the mechanism of creation groups are enriched for genes of the relevant origin. These results are presented in Section S1.1.1 in Additional file 1.

The classification approaches described above designate all genes in a homologous protein family as duplicate and do not attempt to distinguish a single gene as the progenitor of the family. We took this approach, because selecting which gene among a set of duplicates is the ancestral copy is often very difficult - in particular in the case of tandem duplicates [38]. In fact, there is no guarantee that the initial member of the family is still present in the genome. To explore the effect of this choice on our results, we tested another strategy in which we selected the oldest gene from each homologous family (or randomly among the oldest if more than one existed) to serve as the progenitor of the family. The oldest gene was defined as the gene in the family with the most distant ortholog according to the YGOB. For subtelomeric genes, we used the SGD alignments, which each contain a single *S. cerevisiae *gene, to determine the most distant ortholog. This gene was thus assigned to a novel group. Our conclusions held on this adapted classification (Section S1.1.3 in Additional file 1).

### Analysis of interaction network properties

The integration of a protein in the physical interaction network was quantified by its degree (that is, the number of interactions in which it participates) and its betweenness centrality (that is, the fraction of all shortest paths between pairs of other nodes in the network that go through it) [95,96]. Proteins with no interaction data were not considered in the calculation of network statistics.

The number of interactions between proteins in all pairs of age/origin groups was calculated. The significance of the observed number of interactions was quantified by comparing it to the number of interactions between the same groups in 1,000 randomized networks that maintain the degree distribution within groups, but randomize the interactions. An empirical *p*-value for an observed number of interactions was estimated by the proportion of the random networks in which at least as many interactions were observed [97]. Degree-preserving randomizations were performed using a stub-rewiring algorithm [98]. The effect size of the observed difference was quantified using Glass's Δ: the difference between the observed and average number of interactions in the random networks divided by the standard deviation of the number seen in the random networks.

## Abbreviations

AA: amino acid; DIP: database of interacting proteins; GO: gene ontology; ORF: open reading frame; PPI: protein-protein interaction; PPOD: Princeton protein orthology database; SGD: *Saccharomyces *genome database; WGD: whole-genome duplication; YGOB: yeast gene order browser.

## Authors' contributions

JAC conceived the study and carried out the experiments. JAC, KSP, and MS designed the experiments, interpreted the results, and wrote the paper. All authors have read and approved the final manuscript.

## Supplementary Material

Additional file 1 **Supplementary analysis**. This file contains additional analysis and results in support of the main text. Click here for file

Additional file 2 **Classification of *S. cerevisiae *genes into age/origin groups**. This tab-delimited text file contains the classification of all *S. cerevisiae *genes into age/origin groups. Click here for file
