# Supplementary material for: Novel genes exhibit distinct patterns of function acquisition and network integration
Source: Genome Biol. 2010 Dec 27;11(12):R127. doi: 10.1186/gb-2010-11-12-r127 (PMC3046487; doi:10.1186/gb-2010-11-12-r127)
Supplement: Additional file 1 — Supplementary analysis. This file contains additional analysis and results in support of the main text. [file gb-2010-11-12-r127-S1.pdf]

Novel genes exhibit distinct patterns of function acquisition and network integration

Supplemental Material

John A. Capra Katherine S. Pollard Mona Singh

Abstract

This document provides data and analysis in support of the main text. The Robustness section demonstrates that our main findings are robust to the choice of data source and analysis strategy. For several variations of the methods of classifying genes and building interaction networks, we present results that parallel those described in the main text. The Supplemental Analysis section summarizes additional results, including a more fine-grained age classification, that were not included in the article.

S1 Robustness

Our results are built on the analysis of data from several sources of evolutionary and functional information. In the following sections, we demonstrate that our principal conclusions are maintained over a range of data sets and algorithms.

S1.1 Definition of Gene Origin

Our classification of *S. cerervisiae* genes into mechanism of origin groups relies on gene families and evolutionary histories reconstructed across related species. Fully reconstructing these complex sequences of evolutionary events is a very difficult problem; gene loss, fusion, fission, and rearrangement can obscure the origins of a gene. Since this is an area of active research and a variety of methods have been developed for inferring gene families and evolutionary histories, we tested the sensitivity of our conclusions to the use of several computational methods for these tasks.

We investigated two different strategies for gene origin classification. The first approach considers the existence of paralogs of a gene in the same species based on a particular gene family definition. All genes with paralogs in the species are assigned to the `duplicate` category and all other genes are assigned to the `novel` category. The results using families defined by a Jaccard clustering algorithm are presented in the main text, and results on two additional family definitions (from OrthoMCL and InParanoid) are given below. The second approach to origin classification uses predicted evolutionary histories for each family. We take the histories predicted for each gene across 23 fungal species by the Synergy algorithm [42]. Any gene with a duplication on the path from it to the root of the tree or with a homologous orthogroup is assigned to `duplicate`; all other genes are assigned to `novel`. We demonstrate here that the conclusions presented in the main text hold across all these methodological variations with a few minor exceptions.

S1.1.1 Gene Evolutionary History

Synergy

Evolutionary histories and gene trees have been generated for all genes in *S. cerevisiae* by the Synergy algorithm [25, 42]. Synergy builds "orthogroups" of genes derived from a common ancestor by combining analysis of sequence similarity and gene synteny. We downloaded the predicted orthogroups and gene trees from version 1.1 of the Fungal Orthogroups web site on October 19, 2009.

Synergy's predictions were not in complete agreement with those of the family-based method (Figure S1); 76% (4358 of 5770) of the assignments agree. It should also be noted that the Synergy algorithm considered several additional genomes to those used in the ancestral reconstruction of Gordon et al. [39]. However, these differences did not dramatically affect our conclusions. Table S1 demonstrates that young novel genes are still dramatically shorter and less functionally annotated than the other groups. In the Synergy-based analysis, young duplicates are still significantly less essential, less annotated, and less integrated into interaction networks than old duplicates (Table S1). Figure S2 shows that the significant preference for proteins to interact with other proteins of the same age and origin is maintained in this data set.

We also performed the functional and network analysis on the 4358 genes for which the Synergy and Jaccard clustering age/origin assignments agreed. Table S2 and Figure S3 show that these results resemble those observed using either approach alone and support our main conclusions that: young genes are less functionally integrated into the cell than old genes; young novel genes are particularly short and peripheral in function and interactions; and genes in every group are more likely to interact with other genes in the same group than expected.

S1.1.2 Gene Family Definition

The Princeton Protein Orthology Database (PPOD) [40] provides predictions of homologous families from three different algorithms: OrthoMCL [87], MultiParanoid [88], and a Jaccard clustering-based approach. The Jaccard clustering approach was used in the main text because we found it assigned the highest percentage of known WGD duplicates into the same families (85% v. 40–50%). We now give the results for MultiParanoid and OrthoMCL, which are intended to predict smaller orthologous groups across species. In general, the results are similar; however, there are a few differences as a result of the different families predicted by these methods. From our analysis of WGD duplicates, we expect these two other methods to more frequently incorrectly characterize duplicate genes as novel than the Jaccard clustering approach.

OrthoMCL

The main conclusions of our analysis are all supported when gene families from OrthoMCL are used in the age/origin assignment. One notable difference is that the average length of young novel genes is noticeably longer than when the Jaccard clustering approach is used. We suspect that this is the result of a number of diverged duplicate genes not being recognized as duplicates and thus being included in the `novel` group (Table S3). However, the length of the young novel genes is still significantly less than that of the older novel genes, and all other functional and interaction patterns are maintained. The preference of genes to interact with other genes of the same origin and age is also found in this classification (Figure S4), though the preference observed among young novel genes is not significant (p=0.082).

MultiParanoid

Similarly, the main conclusions of our analysis are supported when gene families from MultiParanoid are used in the age/origin assignment (Table S4, Figure S5). However, in this case, the young duplicate genes are nearly as long as the older duplicates, but as before they have significantly fewer interactions and are far less essential. As for OrthoMCL, this may be the result of diverged duplicates not being recognized and thus being assigned to `novel` groups.

The overall similarity of the results on these independent data sets and classifications strongly supports our conclusions.

S1.1.3 Additional Controls

Effect of Subtelomeric Genes

Subtelomeric regions are very dynamic; they experience a large number of rearrangements and duplications. As a result, the ancestral reconstruction of Gordon et al. [39] did not include these regions. We wanted to consider these genes in our analysis, because many lineage-specific genes appear to be born and amplified in these regions. Since we could not perform the pre-WGD ancestor-based age classification on subtelomeric genes, we aged them using alignments of orthologs generated by the SGD (see Methods in main text).

To demonstrate that the patterns we observe among young novel and young duplicate genes are not specific to those found in subtelomeric regions, we repeated the analysis without these genes. The number of young genes is greatly reduced, but where there is sufficient data, the same patterns are apparent (Table S5, Figure S6).

The enrichment for Gene Ontology functional terms related to environmental response was maintained when subtelomeric young duplicate genes were excluded from the analysis. However, the enrichment for carbohydrate processing genes was lost. This argues that the recent innovation in these functions has been focused in subtelomeric regions. The full list of enriched terms when excluding subtelomeric genes is given in Table S6.

Effect of Essential Genes

Essential proteins have been found to participate in more interactions than non-essential proteins [58, 59]. Since older genes are more likely to be essential than younger genes, we repeated our analysis excluding essential genes to test if these old genes carrying out essential functions are responsible for the increase in interactions observed for older genes in the network. Table S7 and Figure S7 demonstrate that the same relationship between the age of a protein and its cellular context was found without essential genes as when essential genes were considered.

Inference of Ancestral Duplicate Copy

Selecting which gene among a set of duplicates is the ancestral copy is often very difficult—particularly in the case of tandem duplicates [38]. Further complicating this task, there is no guarantee that the initial member of the family is still present in the genome. In our analysis, we dealt with this situation by assigning all genes that had experienced a duplication, the members of each homologous family, to the `duplicate` class.

To explore the effect of this choice on our results, we tested another strategy in which we selected the oldest gene from each a homologous family to serve as the progenitor of the family. The oldest gene was defined as the gene with the most distant homolog in the YGOB (or SGD alignments for subtelomeric genes). If there was more than one oldest gene, a progenitor was selected randomly among them. This gene was assigned to the `novel` class. If more than one oldest gene existed, we selected randomly among them. Table S8 and Figure S8 demonstrate that our conclusions hold on this adapted classification.

S1.2 Protein-Protein Interaction Networks

The results presented in the main text reflect the integration of proteins from each age/origin class into a physical protein-protein interaction network consisting of a combination of interaction data from small-scale experiments and high-throughput studies collected in the Database of Interacting Proteins (DIP) [56]. The next several sections show that these conclusions hold on different interaction datasets.

BioGRID

BioGRID [82] is a repository for protein interaction data. Kim and Marcotte [53] following Batada et al. [89] used specialized filters and confidence measures to build a network combining high-throughput and literature-curated interactions from BioGRID. This network contains fewer interactions for young proteins than DIP, but our conclusions hold on this interaction network as well. Table S9 shows that young genes are less integrated into the network. Figure S9 shows that the preference for proteins to interact with other proteins of the same age and origin is also maintained. However, no interactions between young novel proteins were observed in this filtered network.

High-throughput Only

The presence of interactions inferred from small-scale studies could introduce a bias toward interactions involving well-studied proteins into the network. To test the impact of this potential bias, we analyzed the high-throughput only subnetwork of the Kim and Marcotte [53] network, which is easily divided into a literature-curated interaction set and a set determined by high-throughput experimental methods. We obtained similar results when only interactions determined by high-throughput studies were considered. Most notably, young genes are still less integrated into the network than older genes, and young novel genes are the most peripheral (Table S10). The greater network integration of older proteins does not appear to be an artifact of experimental bias. In this reduced set of interactions, there were no interactions within the young protein groups (Figure S10). Overall, we did not observe a significant difference in the percentage of interactions involving young or novel proteins between the high-throughput and literature-curated sets.

S2 Supplemental Data and Analysis

S2.1 GO Functional Enrichment of Young Genes

In the main text we summarized the results of GO annotation enrichment analysis among the groups of young genes. No significant enrichment was found among the young novel genes, but many terms related to environmental response and carbohydrate processing were enriched among the young duplicate genes. The complete lists of enriched terms from each hierarchy are given in Tables S12–S14. See Section S1.1.3 for a discussion of the impact of subtelomeric genes on functional enrichment.

S2.2 A More Specific Classification of Gene Age

We also considered a more specific temporal classification of the `pre-WGD` genes into two age groups: 1) those created prior to the divergence of *S. cerevisiae* and *Schizosaccharomyces pombe* (`pre-WGD-ancestral`) and 2) those created after this divergence but before the WGD (`pre-WGD-post-pombe`). All genes from the `pre-WGD` age group described in the main text were assigned to either `pre-WGD-ancestral` or `pre-WGD-post-pombe` based on their presence or absence in a homologous family in *S. pombe* [83].

Our main conclusions are maintained on this more specific age grouping. The functional properties of genes in the `pre-WGD-post-pombe` group fall in between the `post-WGD` and `pre-WGD-ancestral` groups (Table S11, Figure S11). This additional temporal data point adds strong support to our conclusion that on average genes gain functions and interactions over time. Similarly, the pattern of genes to preferentially interact with other genes of the same age and mechanism of origin is also maintained under this finer classification. This preference is significant for all groups, except the `pre-WGD-post-pombe/duplicate` proteins which also interact with one another more often than expected by chance, but this effect was not significant ($p = 0.13$).

S3 Supplemental Figures

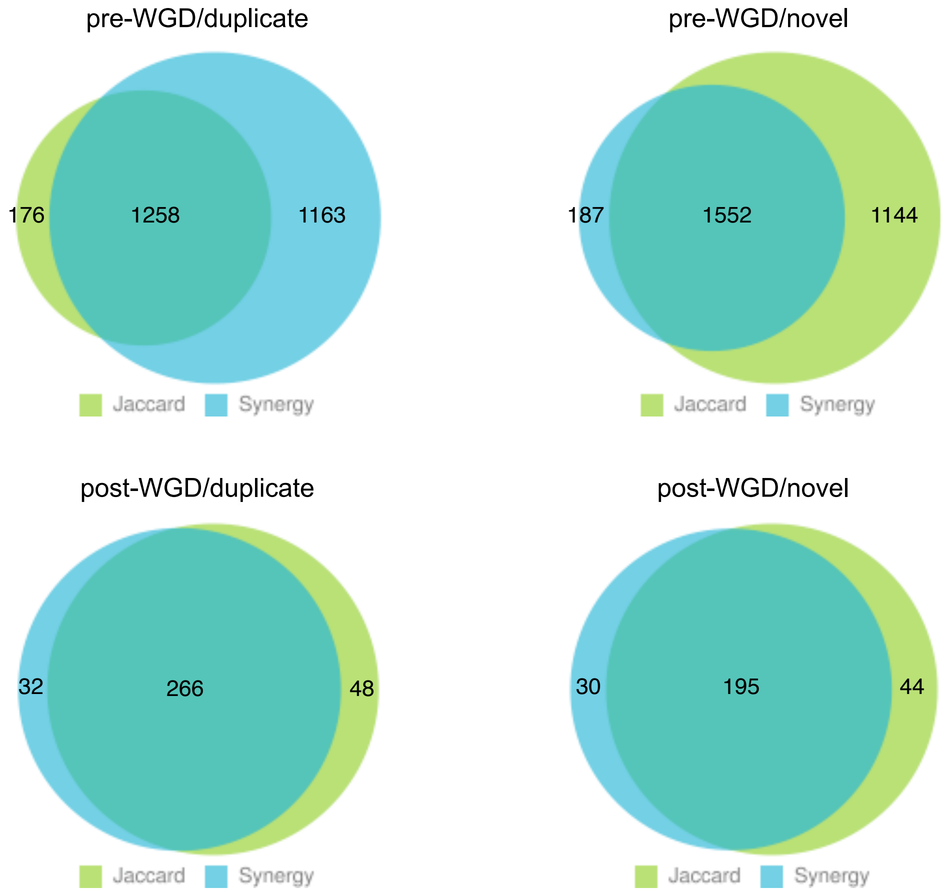

Figure S1: **Overlap of Synergy-based and Jaccard clustering-based age/origin classification.** The mechanism of origin of each gene in *S. cerevisiae* was predicted using the Jaccard clustering family approach and the Synergy gene tree approach. The age of each gene was predicted as described in the main text. WGD proteins are not listed because they did not differ between the classifications.

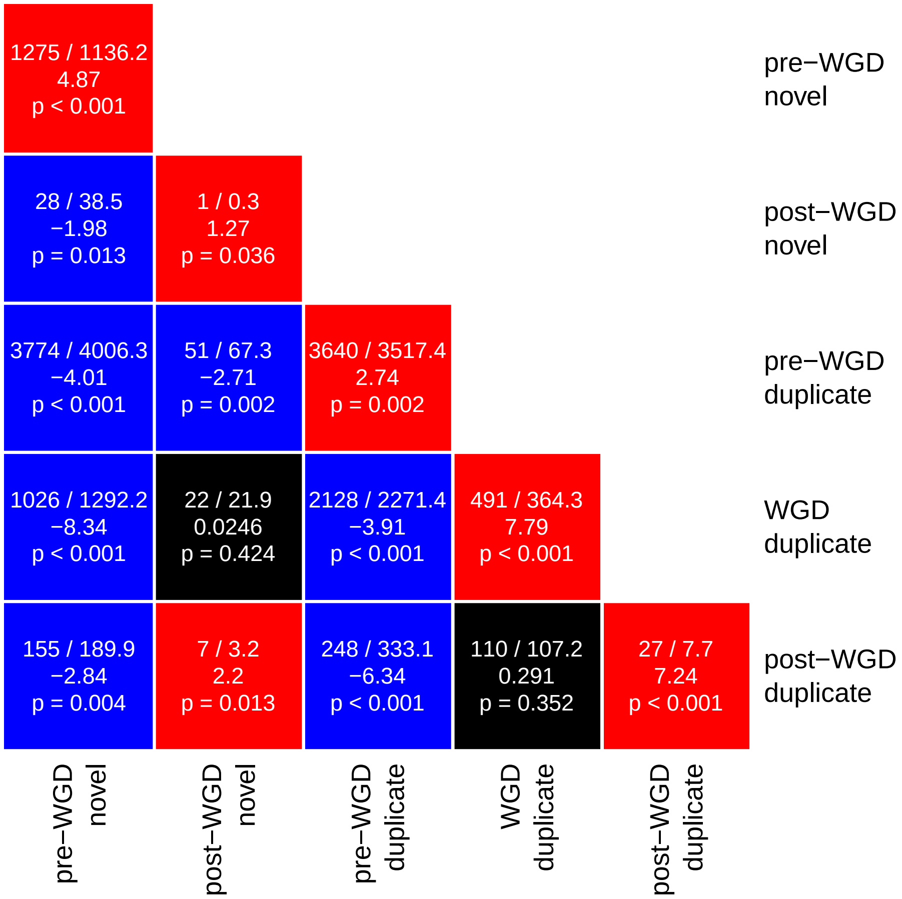

Figure S2: **Significance of interaction preferences when protein origin is predicted from Synergy orthogroups.** All groups and statistics are as in Figure 5 of the main text. As we observed with the age groups used in the main text, the red trend across the diagonal reflects the significant preference for proteins to interact within their age/origin group. The only significant enrichment for interactions between proteins of different age or origin is among young (`post-WGD`) proteins.

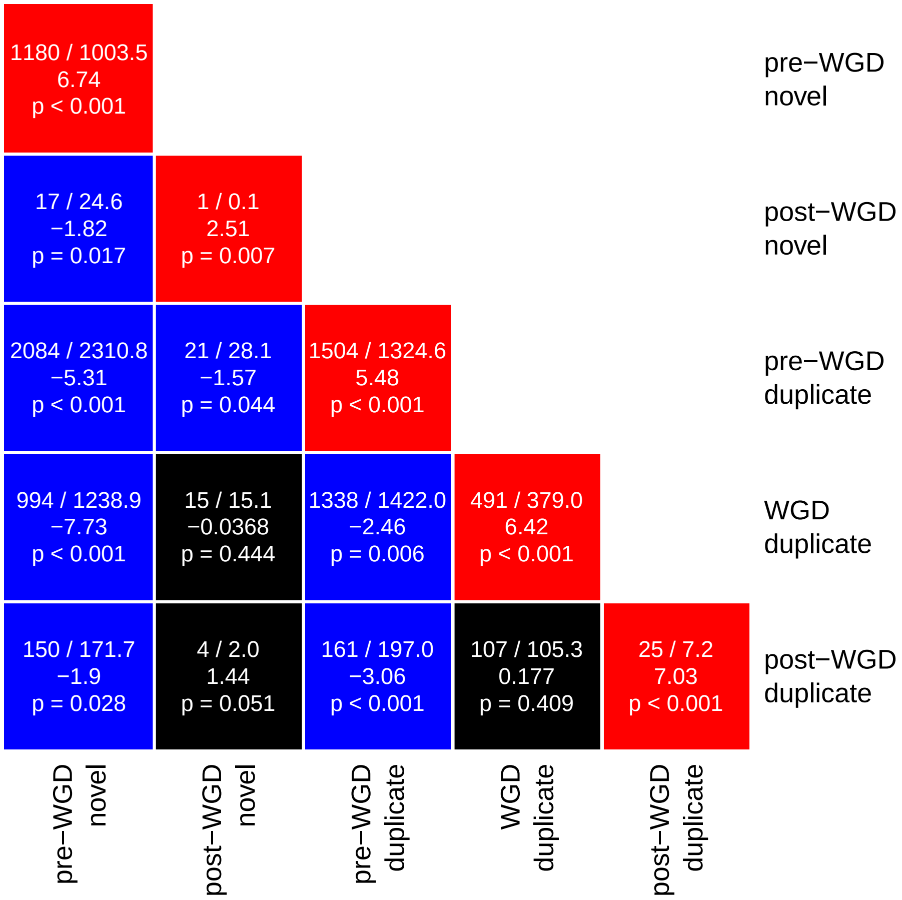

Figure S3: **Significance of interaction preferences when only proteins with agreeing age/origin assignments from Synergy and Jaccard clustering are considered.** All groups and statistics are as in Figure 5 of the main text. As we observed with the age groups used in the main text, the red trend across the diagonal reflects the significant preference for proteins to interact within their age/origin group.

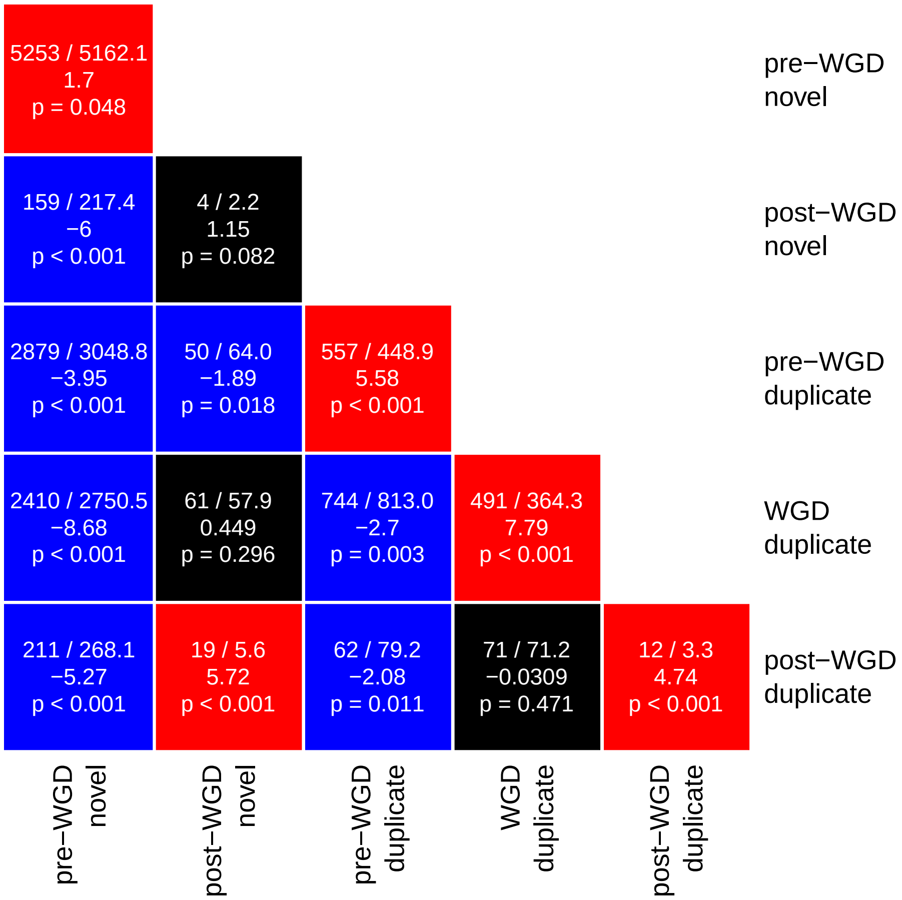

Figure S4: **Significance of interaction preferences by protein age and origin with gene families defined by OrthoMCL.** All groups and statistics are as in Figure 5 of the main text.

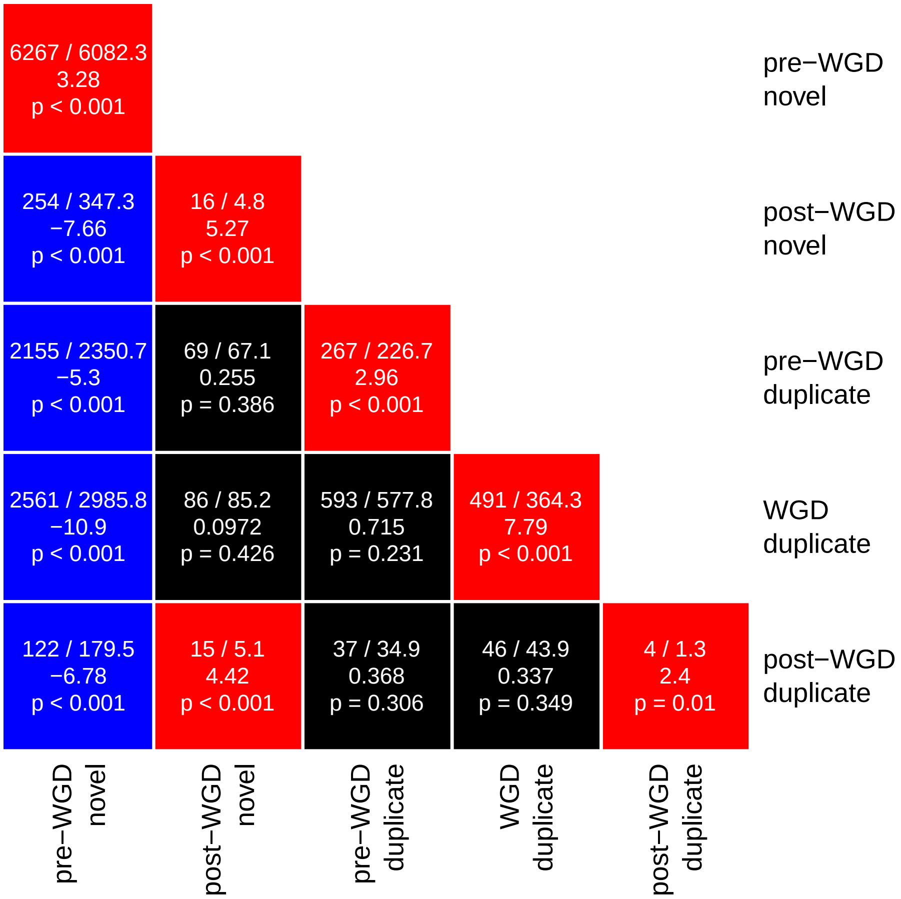

Figure S5: **Significance of interaction preferences by protein age and origin with gene families defined by MultiParanoid.** All groups and statistics are as in Figure 5 of the main text.

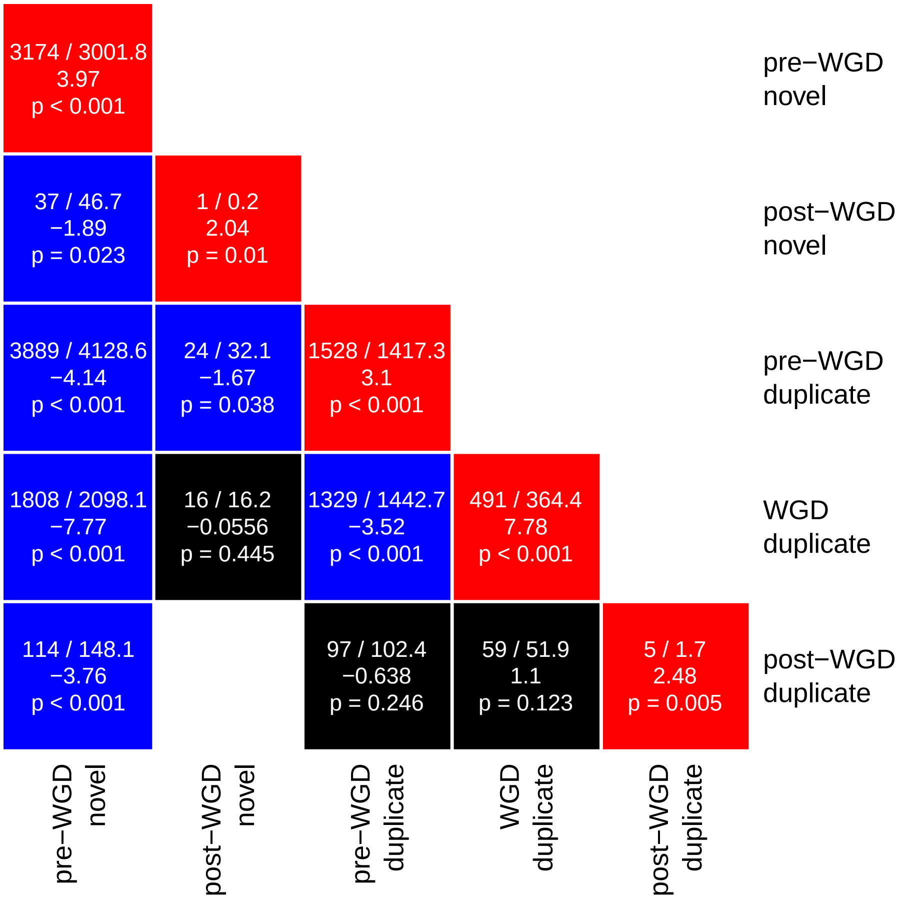

Figure S6: **Significance of interaction preferences by protein age and origin when subtelomeric genes are not considered.** All groups and statistics are as in Figure 5 of the main text. No interactions were observed between non-subtelomeric `post-WGD/novel` and `post-WGD/duplicate` genes.

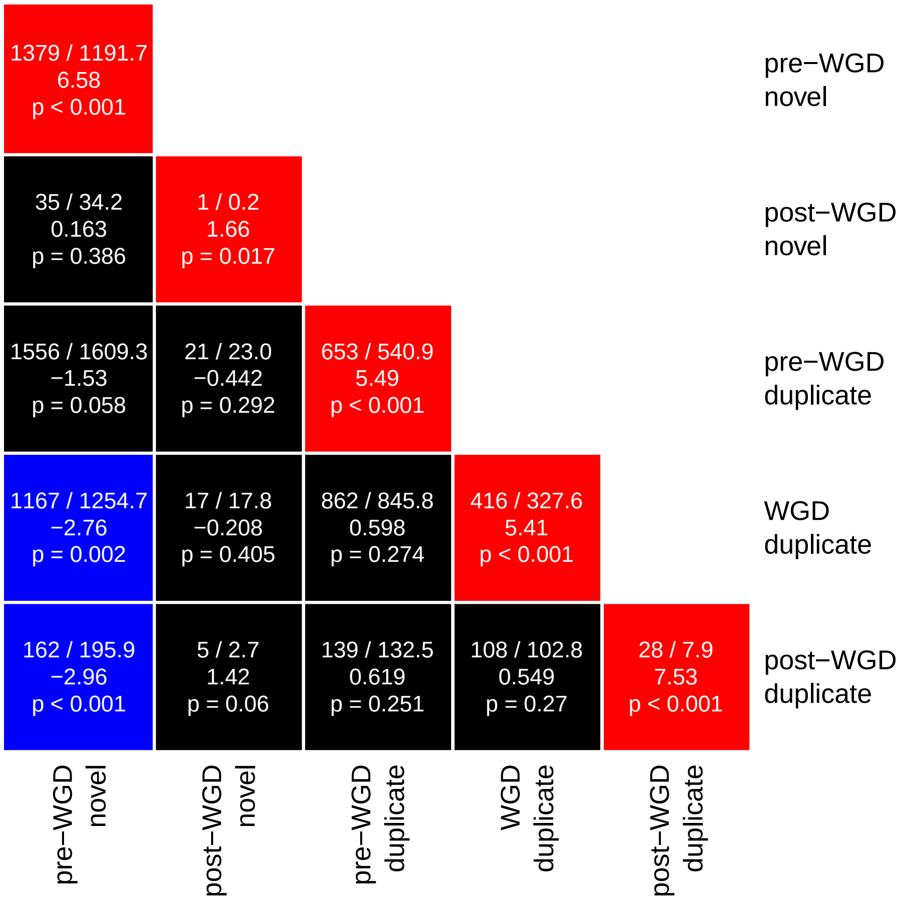

Figure S7: **Significance of interaction preferences by protein age and origin when essential genes are not considered.** All groups and statistics are as in Figure 5 of the main text.

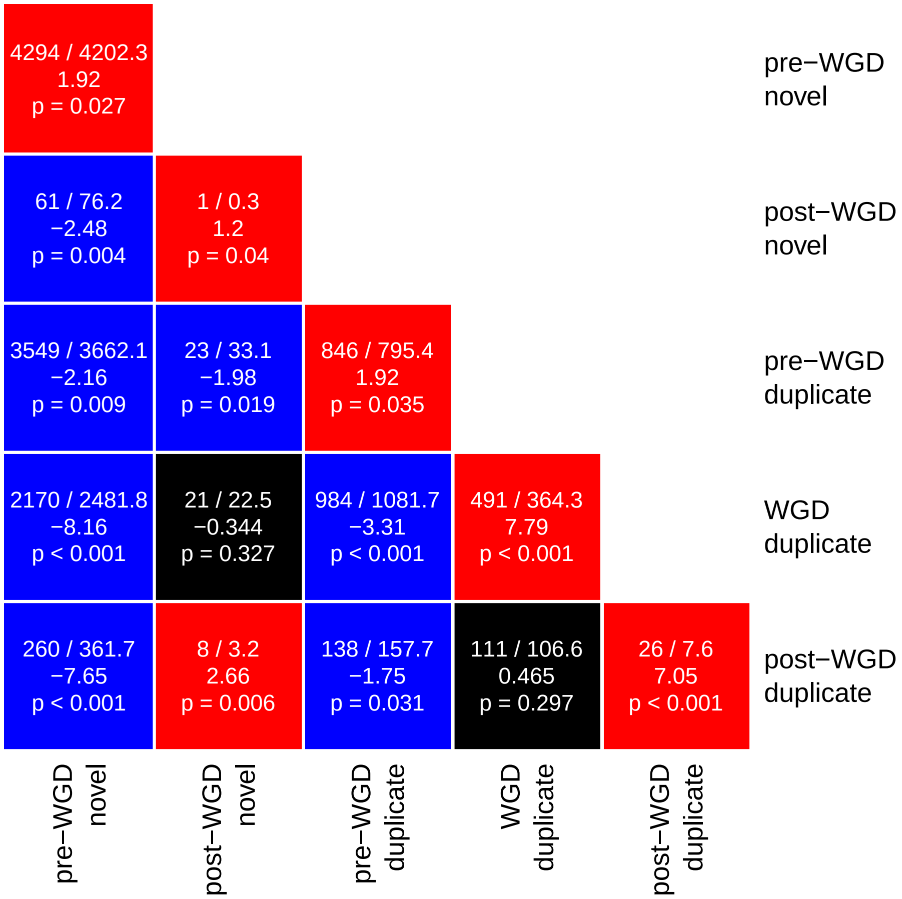

Figure S8: **Significance of interaction preferences by protein age and origin when selecting a progenitor for each gene family.** All other groups and statistics are as in Figure 5 of the main text. As we observed with the age groups used in the main text, the red trend across the diagonal reflects the significant preference for proteins to interact within their age/origin group. The only significant enrichment for interactions between proteins of different age or origin is among young (`post-WGD`) proteins.

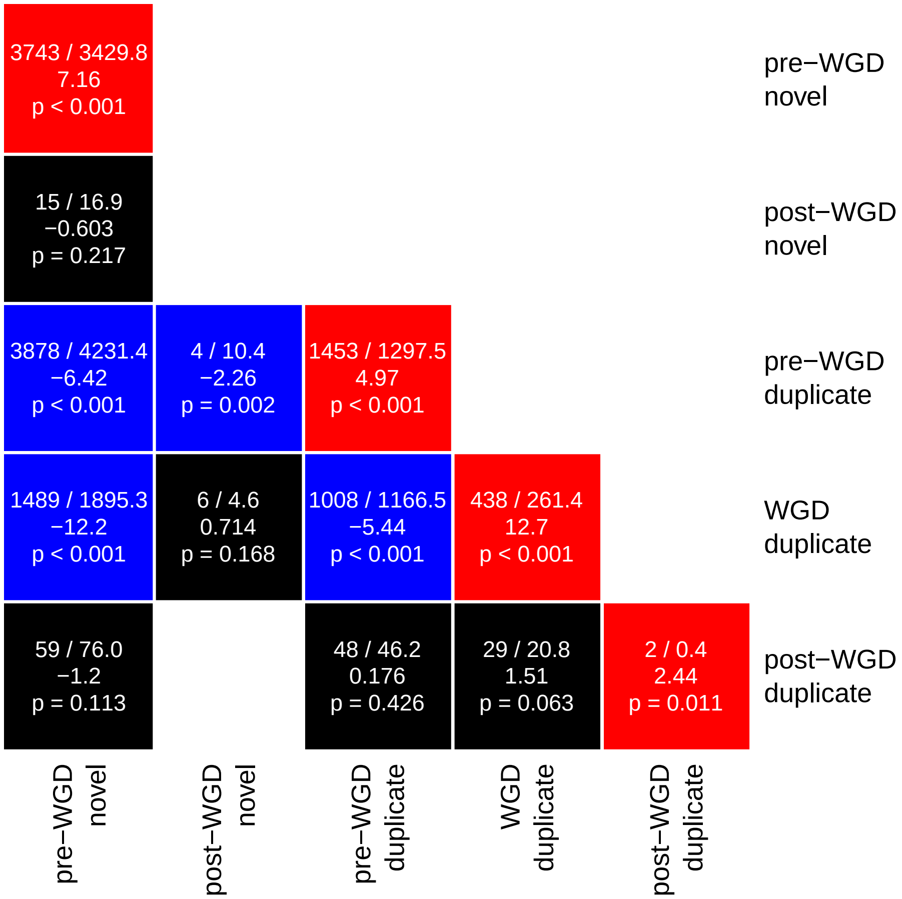

Figure S9: **Significance of interaction preferences by protein age and origin on the filtered BioGRID network.** All groups and statistics are as in Figure 5 of the main text. As we observed with the age groups used in the main text, the red trend across the diagonal reflects the significant preference for proteins to interact within their age/origin group. No interactions were observed within the `post-WGD/novel` group.

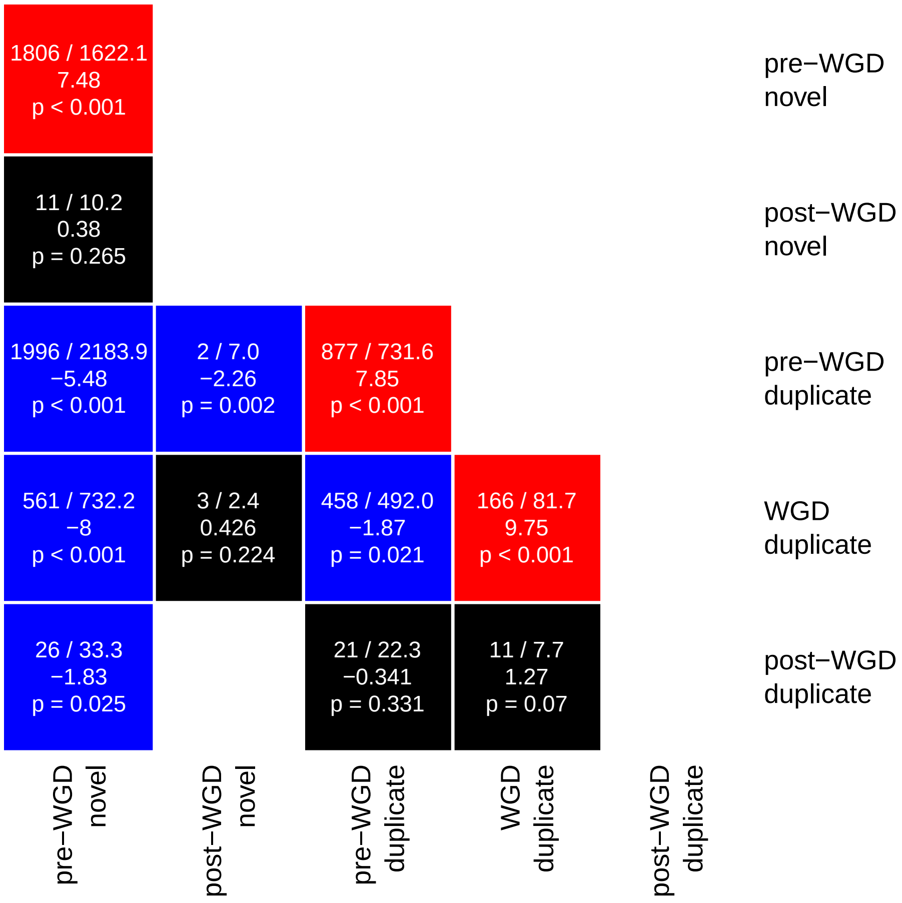

Figure S10: **Significance of interaction preferences by protein age and origin on a network derived from high-throughput studies only.** All groups and statistics are as in Figure 5 of the main text. This reduced interaction set did not contain any interactions of one young protein with another, but the groups for which sufficient interactions were available show the familiar patterns.

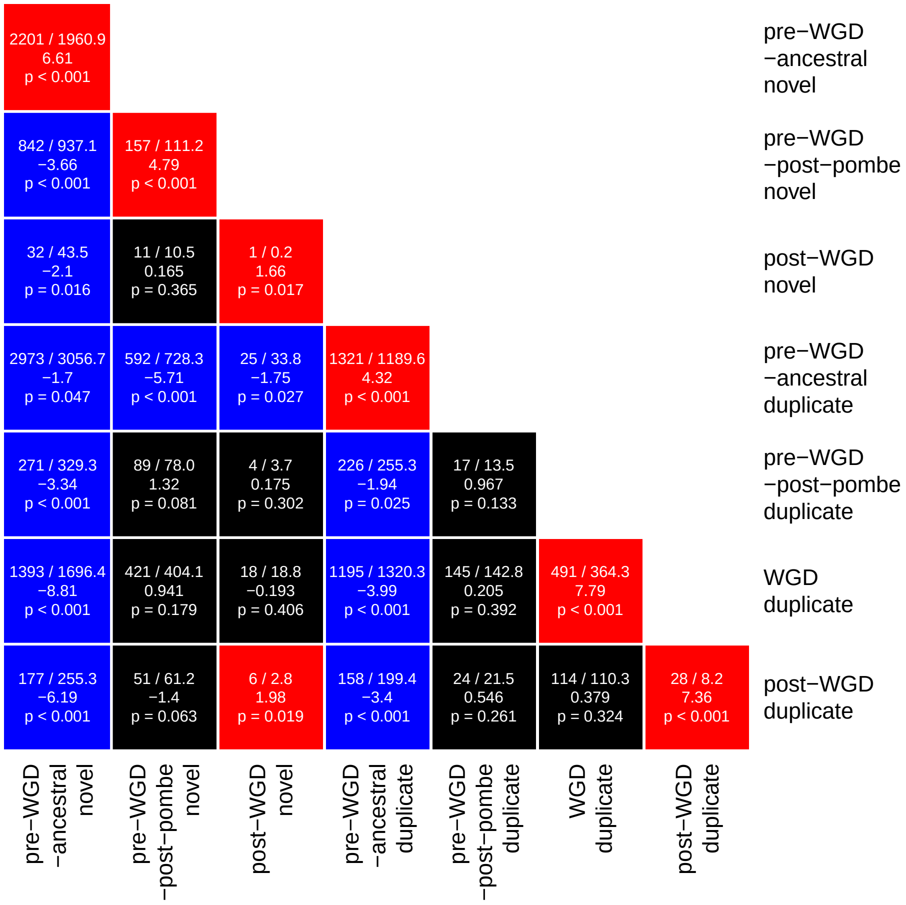

Figure S11: **Significance of interaction preferences by protein age and origin when considering an additional age category.** In this figure, `pre-WGD-post-pombe` genes are those gained prior to the WGD, but after the divergence of *S. cerevisiae* and *S. pombe*. `pre-WGD-ancestral` genes are those gained prior to this divergence. All other groups and statistics are as in Figure 5 of the main text. As we observed in the groups used in the main text, the red trend across the diagonal reflects the significant preference for proteins to interact within their age/origin group. The only significant enrichment for interactions between proteins of different age or origin is among young (`post-WGD`) proteins. We observed more interactions between the `pre-WGD-post-pombe/duplicate` genes than expected, but the effect does not pass the significance threshold (p = 0.133).

S4 Supplemental Tables

Origin	Age	Protein Length	Domain Coverage	Fraction Essential	GO MF Coverage	Degree	Betweenness Centrality
novel	pre-WGD	419.2	0.40	0.293	0.66	5.7	0.000723
novel	post-WGD	259.3	0.15	0.000	0.26	2.0	0.0002
duplicate	pre-WGD	592.0	0.46	0.299	0.80	7.0	0.000958
duplicate	WGD	565.7	0.45	0.075	0.76	5.5	0.000809
duplicate	post-WGD	457.0	0.49	0.017	0.54	3.6	0.000413

Table S1: **Average functional and interaction properties for age/origin gene groups when Synergy orthogroups and gene trees are used to define the origin categories.**

Origin	Age	Protein Length	Domain Coverage	Fraction Essential	GO MF Coverage	Degree	Betweenness Centrality
novel	pre-WGD	415.4	0.39	0.309	0.65	5.0	0.00103
novel	post-WGD	218.9	0.06	0.000	0.22	1.6	0.000209
duplicate	pre-WGD	572.7	0.58	0.318	0.90	6.6	0.00148
duplicate	WGD	573.5	0.45	0.079	0.77	4.6	0.00096
duplicate	post-WGD	478.7	0.52	0.022	0.62	3.6	0.000578

Table S2: **Average functional and interaction properties for age/origin gene groups when only proteins with agreeing Synergy and Jaccard group assignments are considered.**

Origin	Age	Protein Length	Domain Coverage	Fraction Essential	GO MF Coverage	Degree	Betweenness Centrality
novel	pre-WGD	493.0	0.40	0.259	0.67	6.1	0.000782
novel	post-WGD	305.8	0.21	0.024	0.32	2.4	0.000209
duplicate	pre-WGD	539.1	0.58	0.305	0.84	8.2	0.00123
duplicate	WGD	516.3	0.46	0.067	0.73	5.5	0.000809
duplicate	post-WGD	337.7	0.39	0.020	0.31	4.3	0.000572

Table S3: **Average functional and interaction properties for age/origin gene groups with gene families defined by OrthoMCL.**

Origin	Age	Protein Length	Domain Coverage	Fraction Essential	GO MF Coverage	Degree	Betweenness Centrality
novel	pre-WGD	489.8	0.41	0.285	0.67	6.4	0.000842
novel	post-WGD	257.6	0.16	0.032	0.18	3.3	0.000409
duplicate	pre-WGD	561.8	0.58	0.166	0.88	6.9	0.000984
duplicate	WGD	516.3	0.46	0.067	0.73	5.5	0.000809
duplicate	post-WGD	541.6	0.68	0.000	0.82	2.9	0.000273

Table S4: **Average functional and interaction properties for age/origin gene groups with gene families defined by MultiParanoid.**

Origin	Age	Protein Length	Domain Coverage	Fraction Essential	GO MF Coverage	Degree	Betweenness Centrality
novel	pre-WGD	478.3	0.36	0.267	0.62	6.0	0.00077
novel	post-WGD	124.1	0.04	0.024	0.04	1.9	0.000184
duplicate	pre-WGD	549.0	0.57	0.282	0.87	7.4	0.0011
duplicate	WGD	516.3	0.46	0.067	0.73	5.4	0.000807
duplicate	post-WGD	341.0	0.39	0.034	0.47	4.0	0.000439

Table S5: **Average functional and interaction properties for age/origin gene groups when subtelomeric genes are not considered.**

GO Term	Frequency in Group	Background	P-value	FDR
asparagine catabolic process (BP)	4 / 122	5 / 5797	0.00023	0.00
cellular response to nitrogen levels (BP)	4 / 122	6 / 5797	0.00068	0.00
cellular response to nitrogen starvation (BP)	4 / 122	6 / 5797	0.00068	0.00
asparagine metabolic process (BP)	4 / 122	8 / 5797	0.00307	0.00
cellular amino acid catabolic process (BP)	7 / 122	40 / 5797	0.00405	0.00
amine catabolic process (BP)	7 / 122	43 / 5797	0.00664	0.00
cell wall-bounded periplasmic space (CC)	6 / 122	9 / 5797	2.45e-07	0.00
periplasmic space (CC)	6 / 122	9 / 5797	2.45e-07	0.00
external encapsulating structure (CC)	9 / 122	98 / 5797	0.00785	0.03
cell wall (CC)	9 / 122	98 / 5797	0.00785	0.02
fungal-type cell wall (CC)	9 / 122	98 / 5797	0.00785	0.02
asparaginase activity (MF)	4 / 122	6 / 5797	0.00021	0.00

Table S6: Enriched GO functional terms among `post-WGD/duplicate`, non-subtelomeric genes. Frequency in Group gives the fraction of these genes annotated with the given term. Background Frequency gives the overall frequency with which this term is observed across all yeast genes. P-value and false discovery rate (FDR) were computed by the GO:TermFinder tool [60].

Origin	Age	Protein Length	Domain Coverage	Fraction Essential	GO MF Coverage	Degree	Betweenness Centrality
novel	pre-WGD	462.1	0.34	0.000	0.56	4.1	0.00117
novel	post-WGD	143.1	0.05	0.000	0.08	1.5	0.000258
duplicate	pre-WGD	532.4	0.58	0.000	0.85	4.9	0.00146
duplicate	WGD	507.5	0.46	0.000	0.72	4.4	0.00129
duplicate	post-WGD	454.1	0.44	0.000	0.49	3.2	0.000725

Table S7: **Average functional and interaction properties for age/origin gene groups when essential genes are not considered.**

Origin	Age	Protein Length	Domain Coverage	Fraction Essential	GO MF Coverage	Degree	Betweenness Centrality
novel	pre-WGD	481.5	0.39	0.261	0.64	6.1	0.000778
novel	post-WGD	166.6	0.07	0.018	0.09	1.7	0.000147
duplicate	pre-WGD	560.1	0.58	0.285	0.88	7.7	0.00111
duplicate	WGD	516.3	0.46	0.067	0.73	5.5	0.000809
duplicate	post-WGD	459.7	0.47	0.026	0.54	3.9	0.000458

Table S8: **Average functional and interaction properties for age/origin gene groups when a progenitor was selected for each family.**

Origin	Age	Degree	Degree / Length	Betweenness Centrality (BC)	BC / Length
novel	pre-WGD	7.4	0.0212	0.00089	2.16×10^{-6}
novel	post-WGD	1.7	0.00739	1.38×10^{-5}	7.79×10^{-8}
duplicate	pre-WGD	8.2	0.0219	0.00122	3.12×10^{-6}
duplicate	WGD	5.6	0.0124	0.000853	1.81×10^{-6}
duplicate	post-WGD	2.5	0.00599	0.000442	7.54×10^{-7}

Table S9: **Average integration into the filtered BioGRID interaction network by age/origin group.**

Origin	Age	Degree	Degree / Length	Betweenness Centrality (BC)	BC / Length
novel	pre-WGD	5.1	0.0145	0.00119	2.73×10^{-6}
novel	post-WGD	1.5	0.0061	0.000211	1.61×10^{-6}
duplicate	pre-WGD	6.1	0.0168	0.00168	4.12×10^{-6}
duplicate	WGD	3.6	0.00701	0.000998	1.76×10^{-6}
duplicate	post-WGD	1.8	0.00426	0.000378	7.8×10^{-7}

Table S10: **Average integration into the high-throughput experiment only interaction network by age/origin group.**

Origin	Age	Protein Length	Domain Coverage	Fraction Essential	GO MF Coverage	Degree	Betweenness Centrality
novel	pre-WGD-ancestral	498.8	0.44	0.344	0.69	6.7	0.000877
novel	pre-WGD-post-pombe	429.5	0.18	0.079	0.44	4.0	0.000467
novel	post-WGD	143.1	0.05	0.020	0.07	1.8	0.000169
duplicate	pre-WGD-ancestral	552.8	0.58	0.312	0.87	7.8	0.00113
duplicate	pre-WGD-post-pombe	502.2	0.53	0.050	0.83	4.5	0.000552
duplicate	WGD	516.3	0.46	0.067	0.73	5.5	0.000809
duplicate	post-WGD	451.0	0.44	0.024	0.50	3.7	0.000425

Table S11: **Average functional and interaction properties for age/origin gene groups with an additional age category.** In this table, `pre-WGD-post-pombe` genes are those gained prior to the WGD, but after the divergence of *S. cerevisiae* and *S. pombe*. `pre-WGD-ancestral` genes are those gained prior to this divergence. The `WGD/duplicate` genes are not necessarily expected to follow the temporal patterns of other `duplicate` genes as the pressures following the WGD were likely very different than following a small-scale duplication.

GO Biological Process Term	Frequency in Group	Background Frequency	P-value	FDR
carbohydrate transport	11 / 296	37 / 5797	0.00041	0
monosaccharide transport	9 / 296	25 / 5797	0.00064	0
hexose transport	9 / 296	25 / 5797	0.00064	0
telomere maintenance via recombination	7 / 296	19 / 5797	0.00774	0
cellular response to nitrogen levels	4 / 296	5 / 5797	0.00992	0
asparagine catabolic process	4 / 296	5 / 5797	0.00992	0
cellular response to nitrogen starvation	4 / 296	5 / 5797	0.00992	0

Table S12: Enriched GO Biological Process terms in `post-WGD/duplicate`. Frequency in Group gives the fraction of genes in `post-WGD/duplicate` annotated with the given term. Background Frequency gives the overall frequency with which this term is observed across all yeast genes. P-value and false discovery rate (FDR) were computed by the GO:TermFinder tool [60].

GO Molecular Function Term	Frequency in Group	Background Frequency	P-value	FDR
sugar transmembrane transporter activity	11 / 296	21 / 5797	1.30×10^{-07}	0
carbohydrate transmembrane transporter activity	11 / 296	25 / 5797	1.37×10^{-06}	0
glucose transmembrane transporter activity	9 / 296	16 / 5797	1.98×10^{-06}	0
monosaccharide transmembrane transporter activity	9 / 296	17 / 5797	4.04×10^{-06}	0
hexose transmembrane transporter activity	9 / 296	17 / 5797	4.04×10^{-06}	0
mannose transmembrane transporter activity	8 / 296	15 / 5797	2.25×10^{-05}	0
fructose transmembrane transporter activity	8 / 296	15 / 5797	2.25×10^{-05}	0
helicase activity	17 / 296	80 / 5797	4.27×10^{-05}	0
aryl-alcohol dehydrogenase activity	6 / 296	8 / 5797	4.93×10^{-05}	0
transmembrane transporter activity	36 / 296	300 / 5797	0.00011	0
acid phosphatase activity	5 / 296	6 / 5797	0.00022	0
transporter activity	41 / 296	375 / 5797	0.00022	0
oxidoreductase activity, acting on the CH-OH group of donors, NAD or NADP as acceptor	14 / 296	72 / 5797	0.0014	0
oxidoreductase activity, acting on CH-OH group of donors	14 / 296	80 / 5797	0.00488	0

Table S13: Enriched GO Molecular Function terms in `post-WGD/duplicate`.

GO Cellular Component Term	Frequency in Group	Background Frequency	P-value	FDR
plasma membrane	37 / 296	281 / 5797	3.36×10^{-06}	0
external encapsulating structure	19 / 296	98 / 5797	1.90×10^{-05}	0
cell wall	19 / 296	98 / 5797	1.90×10^{-05}	0
fungal-type cell wall	19 / 296	98 / 5797	1.90×10^{-05}	0
cell wall-bounded periplasmic space	6 / 296	9 / 5797	6.71×10^{-05}	0
periplasmic space	6 / 296	9 / 5797	6.71×10^{-05}	0

Table S14: Enriched GO Cellular Component terms in `post-WGD/duplicate`.
